# Prevalence and Predictors of Polypharmacy among Korean Elderly

**DOI:** 10.1371/journal.pone.0098043

**Published:** 2014-06-10

**Authors:** Hong-Ah Kim, Ju-Young Shin, Mi-Hee Kim, Byung-Joo Park

**Affiliations:** 1 Korea Institute of Drug Safety and Risk Management (KIDS), Seoul, Korea; 2 Department of Preventive Medicine, College of Medicine, Seoul National University, Seoul, Korea; University of Glasgow, United Kingdom

## Abstract

**Objective:**

Polypharmacy is widespread in the elderly because of their multiple chronic health problems. The objective of this study was to investigate the prevalence and predictors associated with polypharmacy in a nationally representative sample of Korean elderly individuals.

**Methods:**

We used the Korea Health Insurance Review and Assessment Service – National Patient Sample (HIRA-NPS) data from 2010 and 2011. We used information on 319,185 elderly patients (aged 65 years or older) between January 1, 2010 and December 31, 2011 from the HIRA-NPS database. We defined ‘polypharmacy’ as the concurrent use of 6 medications or more per person, ‘major polypharmacy’ as 11 medications or more, and ‘excessive polypharmacy’ as 21 medications or more. The frequency and proportion (%) and their 95% confidence intervals were presented according to the polypharmacy definition. Polypharmacy was visualized by the Quantum Geographic Information Systems (QGIS) program to describe regional differences in patterns of drug use. Multivariate ordinal logistic regression was performed to estimate odds ratios (ORs) and their 95% confidence intervals (CI) to investigate the risk factors for polypharmacy.

**Results:**

Of the Korean elderly studied, 86.4% had polypharmacy, 44.9% had major polypharmacy and 3.0% had excessive polypharmacy. Polypharmacy was found to be primarily concentrated in the Southwest region of the country. Significant associations between polypharmacy and the lower-income Medical Aid population (OR = 1.52, 95% CI 1.47, 1.56) compared with National Health Insurance patients was observed.

**Conclusions:**

Nationwide efforts are needed for managing polypharmacy among Korean elderly patients. In particular, a national campaign and education to promote appropriate use of medicines for the Medical Aid population is needed.

## Introduction

More than half of the world's population will be over the age of 65 by the year 2030 [1]. As in most other countries, the proportion of elderly people in Korea is increasing every year due to decreased birth rates and increased longevity. The elderly population is projected to increase to twice that of children by 2030 and 4 times that of children by 2060 [2]. Studies show that aging can alter practically all pharmacokinetic processes, including absorption, first-pass metabolism, bioavailability, distribution, protein binding, and renal and hepatic clearance. These alterations contribute to an increasing risk of adverse drug reactions [3]. Additionally, the aging of the population and the resulting increase of multiple chronic diseases have led to multiple drug prescriptions and drug-drug interactions [4].

Medication use in older people is a particular public health concern, since the older population have a higher prevalence of multiple drug use, referred to as “polypharmacy” [5], [6]. Polypharmacy is not uniformly defined in the literature, although the concurrent use of 5–6 or more drugs is a frequently used operational definition [7]–[9]. A systematic review published in 2013 noted that polypharmacy has a clearly established strong relationship with negative clinical outcomes [10]. Several previous studies have also reported that polypharmacy is associated with the increased occurrence of adverse drug reactions, drug-drug interactions, inappropriate medication [11]–[13], and poorer health outcomes such as malnutrition, functional impairment, falls, fractures, and hospitalization [14]–[16]. Moreover, several previous studies in Korea have reported the prevalence of polypharmacy in Korean elderly, but the studies suggested that further research be performed to classify polypharmacy categories by the number of drugs and polypharmacy status by regions and predictors (gender, age, health insurance type) [17]–[20]. Therefore, the objective of this study was to investigate the prevalence and regional variation and to evaluate the role of different factors associated with polypharmacy in a nationally representative sample of 319,185 Korean elderly patients.

## Materials and Methods

### Data source

We used the Health Insurance Review and Assessment Service – National Patient Sample (HIRA-NPS) data from 2010 and 2011. Korean healthcare providers have been required to submit claims on medical services to HIRA for review of medical care costs since 2000. Accordingly, the HIRA database contains all medical information for approximately 50 million Koreans. HIRA-NPS consists of 3% of all Korean patients covering 319,185 elderly patients and 100,838,744 prescriptions. The HIRA-NPS database was constructed using gender- and age-stratified random sampling. In order to examine whether this sample data appropriately reflected the population, research was conducted. The representativeness and validity of this sample database has been confirmed by comparing the estimation from the data and the whole population [21].

The HIRA-NPS contains each patient's unique encrypted identification number (ID), age, gender, primary diagnosis, secondary diagnosis, surgical or medical treatment administered, whether the individual was an inpatient or outpatient, type of insurance (National Health Insurance or Medical Aid), medical expenses, medical institution identification number (ID), and prescriptions. The diagnosis was coded according to the International Classification of Disease, Tenth Revision (ICD-10). The generic drug names were coded according to the Korean national code system.

### Study subjects

The study population consisted of elderly outpatients aged 65–99 years who visited clinics and/or hospitals for ambulatory care and received at least one prescription between January 1, 2010 and December 31, 2011. Prescriptions for outpatients aged 65 years or older were included. Prescriptions for cancer patients and veterans were excluded, because they are managed by another health care system by a different insurance type [17].

### Definition and Measure

To measure the number of drugs, we-recoded the Korean national drug code according to the WHO-Anatomical Therapeutic Chemical (ATC) Classification System. The unit of a drug was applied as the 5^th^ ATC level administered. As an indicator of polypharmacy, ‘polypharmacy’ was defined as the concurrent use of 6 medications or more per person [7], [9], [17], [22], [23], ‘major polypharmacy’ as 11 medications or more [7], [9], [24]–[26], and ‘excessive polypharmacy’ as 21 medications or more [7], [26]. The non-polypharmacy group included persons using five or fewer drugs concomitantly. These cut-off points were chosen based on previous studies and current treatment patterns of elderly populations. Reflecting the expanding opportunities for drug treatment of elderly patients, we chose a higher cut-off point for polypharmacy. In the definition for polypharmacy, we include the situation in which a patient received more than one prescription concurrently in order to investigate the maximum number of drugs administered per patient.

We defined frequent conditions among elderly patients according to previous studies [19], [27]. The ICD-10 codes for these conditions were grouped in order to account for coding variance among physicians for the same syndrome. The result of this procedure was a list of 51 single codes and code groups further referred to as ‘chronic conditions’ (see Table S1).

The analysis of regional distribution was based on a total of 16 districts: Seoul, Busan, Incheon, Daegu, Gwangju, Daejeon, Ulsan, Gyeonggi-do, Gangwon-do, Chungcheongbuk-do, Chungcheongnam-do, Jeollabuk-do, Jeollanam-do, Gyeongsangbuk-do, Gyeongsangnam-do, and Jeju-do. We classified the types of health insurance as National Health Insurance and Medical Aid. As a compulsory social insurance program, Korean health insurance covers the whole population living in the country. If patients had an income less than the legal minimum cost of living, they were eligible for Korean Medical Aid [28].

To investigate the relation between the number of drugs per patient and the frequency of medical institution visits per patient, we found the total number of visits to different healthcare organization per patient during the study period; that is, how frequently the patient visited healthcare facilities during the study period. This referred to all ambulatory care visits – primary clinics, secondary facilities, and tertiary facilities - for the medications. These patterns are displayed in a cross-table that matches the number of drugs per person and the frequency of medical institution visits.

### Statistical analysis

To estimate the prevalence of polypharmacy, the frequency and proportion (%) and their 95% confidence intervals were presented for each operational definition. The age-standardized prevalence was calculated reflecting the region's specific demographic distribution. Regional differences in polypharmacy were visualized by Quantum Geographic Information Systems (QGIS) (OSGeo, Beaverton, OR, USA). This geographic software effectively presents the nationwide drug use pattern. The total number of visits to different healthcare organizations were compared between the non-polypharmacy and polypharmacy group. The p-value was calculated by using the ANOVA test for continuous variables and chi-squared test for categorical variables.

Logistic regression was performed to estimate the odds ratios (ORs) and their 95% confidence intervals (CI) to investigate predictors for polypharmacy. We also performed multivariate ordinal logistic regression to investigate the overall effects on the predictors of polypharmacy. Possible predictors included the gender, age, health insurance type (National Health Insurance, Medical Aid), number of chronic conditions, and type of chronic conditions. All statistical analyses were performed using SAS 9.3 (SAS Institute Inc., Cary, NC, USA). A 2-tailed value of P<0.05 was considered statistically significant.

### Ethics statement

The study protocol was approved by the Institutional Review Board of the Korea Institute of Drug Safety and Risk Management. Obtaining informed consent from the study population was waived by the board.

## Results

Among the 319,185 patients, 21,383 (6.7%) were aged ≥85 years, 96,400 (30.2%) were aged 75–84 years, and 201,402 (63.1%) were aged 65–74 years. Females composed 60.0% of the total (Table 1). During the study year, an 86.4% estimated prevalence of polypharmacy was found, while 143,218 (44.9%; 95% CI 44.6, 45.0) had major polypharmacy and 9,669 (3.0%; 95% CI 2.7, 3.4) had excessive polypharmacy (Table 2).

**Table 1:** General characteristics of Korean elderly patients in 2010 and 2011.

Characteristics	Number	%
Gender		
Male	127,626	40
Female	191,559	60
Age	Mean±SD	73.7±20.0
	(Min, Max)	(65, 999)
65–69	108,189	33.9
70–74	93,213	29.2
75–79	62,246	19.5
80–84	34,154	10.7
85+	21,383	6.7
Health insurance type		
National Health Insurance	291,250	91.2
Medical Aid	27,935	8.8
Number of chronic conditions	Mean±SD	6.98±4.03
	(Min, Max)	(0, 31)
Diagnostic code of chronic conditions		
Chronic gastritis/GERD	241,617	75.7
Hypertension	189,305	59.3
Chronic low back pain	151,209	47.4
Diseases of the skin and subcutaneous tissue	136,620	42.8
Allergies	128,202	40.2
Osteoarthrosis	117,563	36.8
Lipid metabolism disorders	103,672	32.5
Rheumatoid arthritis/Chronic polyarthritis	94,756	29.7
Diabetes mellitus	85,044	26.6
Atherosclerosis/PAOD	66,628	20.9

SD = Standard deviation.

(GERD, Gastroesophageal reflux disease; PAOD, peripheral arterial occlusive disease).

**Table 2:** Prevalence of polypharmacy, major polypharmacy, and excessive polypharmacy among Korean elderly (aged ≥65 years) subjects (total 319,185).

Category	Number	%	95% Confidence Interval
Polypharmacy (≥6 drugs)	275,881	86.4	86.3 to 86.6
Major polypharmacy (≥11 drugs)	143,218	44.9	44.6 to 45.0
Excessive polypharmacy (≥21 drugs)	9,669	3.0	2.7 to 3.4

**Figure 1:**
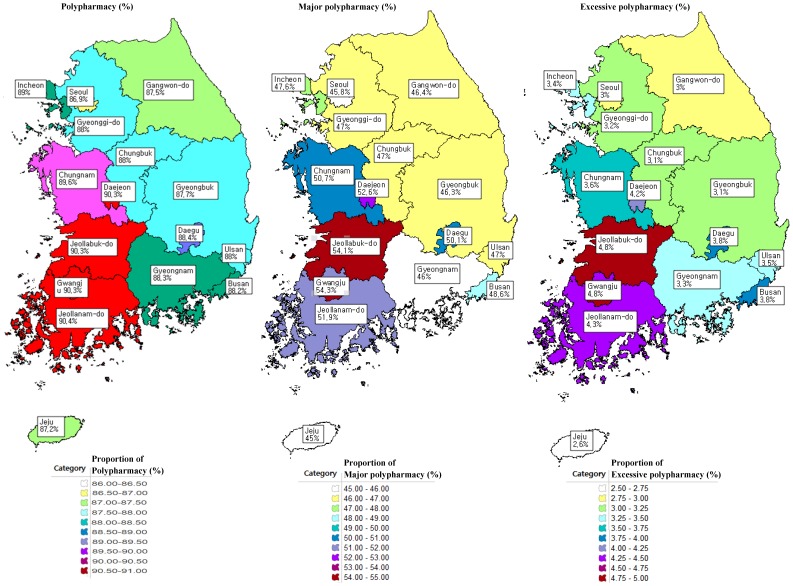
Regional distribution of polypharmacy (≥6, ≥11, ≥21) by number of simultaneous drugs among Korean elderly patients.

Polypharmacy is highly prevalent in the Southwest rural region of the country. The highest age-adjusted polypharmacy prevalence rate was 90.4% in Jeollanam-do, and the lowest was the district of Seoul at 86.9% (Figure 1). The mean number of visits to different healthcare organizations was 1.88 (±1.16) in the non-polypharmacy group, 7.50 (±3.73) in the excessive polypharmacy group (p-value<0.001). Most non-polypharmacy individuals (98.7%) visited less than 5 healthcare institutions. Moreover, only 1.3% of non-polypharmacy group visited over 6 healthcare institutions, but more than half of excessive polypharmacy group visited 6 or more healthcare institutions (Table 3).

**Table 3:** The number of drugs per person and the frequency of ambulatory care visits per person during the study period.

		1–5		6–10		11–20		21+		
		Number	%	Number	%	Number	%	Number	%	P-value*
Number of visits to different healthcare organizations	1–5	42,728	98.7	110,133	83.0	75,458	56.5	3,156	32.6	<.001
	6–10	572	1.3	21,601	16.3	50,467	37.8	4,666	48.3	<.001
	11–20	4	0.0	928	0.7	7,565	5.7	1,801	18.6	<.001
	21+	0	0.0	1	0.0	59	0.0	46	0.5	<.001
	Total	43,304	100	132,663	100	133,549	100	9,669	100	<.001

^*^ *P*-value by ANOVA test for continuous variables and chi-squared test for categorical variables.

Predictors for polypharmacy were being male, part of the 70–84 age group, having Medical Aid, and a larger number of chronic conditions. Notably, Medical Aid was associated with polypharmacy (OR 1.52; 95 CI 1.47, 1.56) after adjusting for gender, age, and chronic conditions (Table 4).

**Table 4:** Logistic regression analysis of predictors of polypharmacy among Korean elders.

	[OR (95% CI)] (modeling the likelihood of being classified into a higher level of polypharmacy)	[OR (95% CI)] (Reference, non-polypharmacy group)		
Parameter		Polypharmacy		
		6–10	11–20	21+
**Gender**				
Female	1	1	1	1
Male	1.23 (1.21–1.25)	1.05 (1.02–1.08)	1.25 (1.19–1.30)	1.85 (1.54–2.23)
**Age**				
65–69	1	1	1	1
70–74	1.07 (1.05–1.09)	1.04 (1.00–1.07)	1.13 (1.07–1.20)	1.22 (0.98–1.52)
75–79	1.10 (1.08–1.12)	1.03 (0.99–1.07)	1.12 (1.05–1.20)	1.20 (0.93–1.55)
80–84	1.04 (1.02–1.07)	0.90 (0.85–0.94)	1.02 (0.94–1.10)	1.36 (1.01–1.84)
85+	0.87 (0.84–0.90)	0.78 (0.74–0.82)	0.81 (0.74–0.88)	0.69 (0.47–1.03)
**Health insurance type**				
National Health Insurance	1	1	1	1
Medical Aid	1.52 (1.47–1.56)	1.09 (1.03–1.16)	1.60 (1.47–1.75)	3.61 (2.73–4.76)
**Number of chronic conditions**	1.52 (1.52–1.53)	1.65 (1.63–1.67)	2.48 (2.44–2.53)	3.33 (3.14–3.52)
**Chronic conditions**				
Chronic gastritis/GERD	2.19 (2.15–2.24)	2.00 (1.94–2.06)	3.03 (2.89–3.19)	5.34 (4.05–7.04)
Hypertension	1.86 (1.83–1.90)	1.67 (1.62–1.73)	2.99 (2.85–3.14)	3.69 (3.05–4.48)
Chronic low back pain	1.09 (1.07–1.11)	1.16 (1.12–1.21)	1.15 (1.09–1.21)	1.11 (0.92–1.35)
Diseases of the skin and subcutaneous tissue	1.13 (1.11–1.15)	1.52 (1.47–1.57)	1.36 (1.29–1.43)	1.31 (1.09–1.58)
Allergies	1.36 (1.34–1.38)	2.51 (2.41–2.61)	2.39 (2.26–2.53)	1.96 (1.62–2.37)
Osteoarthrosis	1.01 (1.00–1.03)	1.02 (0.98–1.06)	0.98 (0.93–1.04)	0.92 (0.76–1.12)
Lipid metabolism disorders	1.01 (1.00–1.03)	0.85 (0.81–0.89)	0.83 (0.78–0.88)	0.73 (0.60–0.90)
Rheumatoid arthritis/Chronic polyarthritis	1.03 (1.01–1.05)	1.51 (1.44–1.58)	1.32 (1.25–1.40)	0.89 (0.72–1.10)
Diabetes mellitus	1.41 (1.38–1.43)	1.12 (1.07–1.17)	1.51 (1.43–1.61)	1.77 (1.33–2.17)
Atherosclerosis/PAOD	0.93 (0.91–0.95)	0.87 (0.83–0.92)	0.82 (0.76–0.88)	0.78 (0.62–0.98)

(GERD, Gastroesophageal reflux disease; PAOD, peripheral arterial occlusive disease).

## Discussion

This study was performed to describe the patterns of polypharmacy in the elderly using a nationally representative claims database. We found that polypharmacy among the Korean elderly was dramatically higher than that found in elderly subjects surveyed in other studies [7], [29]. Regional variation was observed among the polypharmacy, major polypharmacy, and excessive polypharmacy groups. It is worth noting that polypharmacy occurred in the Medical Aid group after adjusting for gender, age, and chronic conditions.

Several previous studies have also reported the prevalence of polypharmacy. Among them, the results from a Taiwanese study were very similar to our own. Chan et al., reported that 83.5% were categorized as having polypharmacy (6 or more drugs concomitantly) among national samples of 11,338 elders [30]. Chan et al. also reported that the prevalence of polypharmacy and major polypharmacy among disabled Taiwanese elderly patients was 81% and 38%, respectively [24]. However, western countries such as the US or Europe showed a substantially lower proportion than our results. Dwyer et al. in the USA, reported that the prevalence of polypharmacy (9 or more drugs concomitantly) among nursing home residents in 2004 was 39.7% [31]. Jyrkkä et al., in Finland, reported that 28% belonged to the excessive polypharmacy group, and 33% to the polypharmacy group [7].

Compared to the previous research, the prevalence of polypharmacy in Korean elderly patients is much higher than in any other country. In addition, the calculated prevalence might be underestimated because we did not include drugs non-reimbursable by insurance such as over-the-counter medications. Methodological differences also partially explain the observed differences. With a culture that prefers taking medicines and health supplements such as herbal medicines in Asian countries, polypharmacy had been a problem reported in several countries and is considered a serious public health concern [32], [33]. Our research also showed that polypharmacy was more frequent with an increasing number of visits to different healthcare organizations. As the number of drugs per patient increases, the mean number of visits to different healthcare organizations increases. Furthermore, among excessive polypharmacy group patient, there are 46 patients who had visited more than 21 healthcare providers during the study period. Overlapping medications with switching of healthcare providers was previously reported as a serious problem [34].

Several previous studies have also reported the prevalence of polypharmacy in Korean elderly. Among them, Park et al. reported that 51.4% were categorized as having polypharmacy in Korean elderly outpatients [17]. In this study, polypharmacy was defined as the concurrent use of 6 or more oral drug for only one day, excluding injection drugs. The result of this study is different from the prevalence of polypharmacy from our research because of time period. We performed further research based on classifying polypharmacy categories by the number of drugs. We also suggest that regional variation exists by polypharmacy categories and predictors (gender, age, health insurance type) of polypharmacy be investigated. Moreover, we found the positive tendency between the number of drugs per patient and the frequency of ambulatory care visits per patient during the study period.

In our study, the males were found to be more likely to be exposed to polypharmacy. We identified studies that reported a positive correlation between male gender and polypharmacy exposure [7], [24]. Conversely, many studies have reported a correlation between polypharmacy and the female gender [9], [35]–[38]. Such discrepancies among study findings could be due to differences in physicians' prescription attitude toward the genders, as well as to differences between genders in educational and socioeconomic characteristics [39]. Further research exploring the relationship between gender and polypharmacy is warranted.

Our main finding about regional variation and polypharmacy in the Medical Aid population is consistent with some previous research. National Health and Nutrition Examination Survey, III, 1988–1994 (NHANES III) data suggested that polypharmacy may be differentially expressed by geographical region – with the Northeast region of the US having the greatest mean number of concurrently prescribed medications in the US [40]. Insurance status was also associated with polypharmacy. Patents with Medicaid coverage were 3 times more likely to be associated with polypharmacy than those with other sources of payment. This study suggested that the prescription benefits programs under Medicaid insurance plans should be used to reduce polypharmacy through quality improvement measures, such as drug utilization reviews [41].

Our study had several strengths. First, the study population represented Korean elderly patients by using the National Health Insurance claims database. The HIRA-NPS database contains gender- and age-stratified random samples that appropriately reflect the population. The representativeness, reliability, and validity of the database has been confirmed [21]. Second, because the HIRA database included various parameters including medical care utilization status, geographic division, and Korean national drug code, this study was able to provide detailed and precise information on polypharmacy patterns (see Table S2). Third, Korean patients pay a fee-for-service for all healthcare services including drugs; therefore, any misclassification of reimbursable prescription drugs should not have occurred. Lastly, the regional differences in polypharmacy patterns were visualized by a QGIS program reflecting the recent methodological trend of using geographical software to describe regional variations in the public health field. It effectively showed the nationwide drug prescription pattern. Based on the results of this study, feedback to physicians is possible, and it might be a useful tool for improving prescribing practices and nationwide standardization of rational drug prescribing.

The limitations of this study included the following: As with other studies using a claims database retrospectively, not electronic medical records, we were not able to identify adverse effects of individuals due to polypharmacy. Adverse drug reactions arising from polypharmacy should be studied and efforts should be made to minimize serious drug-drug interactions among elderly patients. Moreover, it was difficult to identify whether patients were taking over–the-counter (nonprescription) drugs (such as cough medicines and pain killers) or health functional foods (such as vitamin supplements) not covered by the HIRA. Therefore, we might have underestimated the true prevalence of polypharmacy among Korean elderly patients by not including these medicines. With our definition of polypharmacy, it can include both continuous drug use for chronic diseases (hypertension, lipid metabolism disorder) and short term drug use (such as cough medicines) for minor illness. However, we searched for the maximum number of drugs administered concurrently and suggest a ‘snapshot’ of polypharmacy status in Korean elders. Further study exploring polypharmacy status by time period is needed.

In summary, the results of this study suggest that more efforts will be needed for managing polypharmacy among Korean elderly patients. For management of rational drug use for geriatric public health, population-based prospective cohort studies must be conducted. These efforts lead us to discover longitudinal comprehensive data on health information among Korean elderly patients. Also, a concurrent Drug Utilization Review (DUR) between institutions has been implemented. This system allows doctors to be able to review the medications of their patients, even prescriptions from other medical institutions. As a result, the DUR system prevents doctor shopping, which leads to patients being exposed to inappropriate medications. Moreover, this system helps avert unnecessary polypharmacy, contraindicated drug-drug interaction, therapeutic duplication drug use and overlapping drug use of the same pharmacological classes. For elderly patients, especially the Medical Aid group, a national campaign and education ‘notify to doctors of all the medicines you take’, will be needed. Along with this national campaign and education, additional education for health care professionals on reducing unnecessary polypharmacy will also be needed.

## Supporting Information

Table S1 List of the 51 chronic conditions used in this study and their ICD codes.
(DOCX)

Table S2 The number of drug users in ATC Classification according to polypharmay status in elderly.
(DOCX)
