# Supplementary material for: Prevalence and Predictors of Polypharmacy among Korean Elderly
Source: PLoS One. 2014 Jun 10;9(6):e98043. doi: 10.1371/journal.pone.0098043 (PMC4051604; doi:10.1371/journal.pone.0098043)
Supplement: Table S1 — List of the 51 chronic conditions used in this study and their ICD codes. (DOCX) [file pone.0098043.s001.docx]

**Table S1. List of the 51 chronic conditions used in this study and their ICD codes**

| **No.** | **Chronic condition** | **ICD-10 codes** |
| --- | --- | --- |
| 1 | Hypertension | I10, I11, I12, I13, I14, I15 |
| 2 | Cerebral ischemia/Chronic stroke | I60, I61, I62, I63, I64, I69, G45 |
| 3 | Lipid metabolism disorders | E78 |
| 4 | Chronic ischemic heart disease | I20, I21, I25 |
| 5 | Cardiac valve disorders | I34, I35, I36, I37 |
| 6 | Cardiac arrhythmias | I44, I45, I46, I47, I48, I49 |
| 7 | Cardiac insufficiency | I50 |
| 8 | Diabetes mellitus | E10, E11, E12, E13, E14 |
| 9 | Thyroid dysfunction | E01, E06, E07 |
| 10 | Rheumatoid arthritis/  Chronic polyarthritis | M05, M06, M79 |
| 11 | Osteoarthrosis | M15, M16, M17, M18, M19 |
| 12 | Osteoporosis | M80, M81, M82 |
| 13 | Chronic low back pain | M40, M41, M42, M43, M44, M45, M47, M48.0, M48.1, M48.2, M48.5, M48.6, M48.7, M48.8, M48.9, M50, M51, M52, M53, M54 |
| 14 | Chronic bronchitis/Emphysema | J40, J41, J42, J43 |
| 15 | Asthma/COPD | J44, J45, J47 |
| 16 | Tuberculosis | A15, A16, A17, A18 |
| 17 | Cataract | H25, H26, H28, Q12 |
| 18 | Glaucoma | H40, H42 |
| 19 | Chronic otitis media | H65.2, H65.3, H66.1, H66.2 |
| 20 | Chronic gastritis/GERD | K21, K25, K26, K27, K28, K29.2, K29.3, K29.4, K29.5, K29.6, K29.7, K29.8, K29.9 |
| 21 | Liver disease | K70, K71.3, K71.4, K71.5, K71.7, K72.1, K72.9, K73, K74, K76 |
| 22 | Renal insufficiency | N18, N19 |
| 23 | Prostatic hyperplasia | N40 |
| 24 | Urinary incontinence | N39.3, N39.4, R32 |
| 25 | Anemia | D50, D51, D52, D53, D55, D56, D57, D58, D59.0, D59.1, D59.2, D59.4, D59.5, D59.6, D59.7, D59.8, D59.9, D60.0, D60.8, D60.9, D61, D63, D64 |
| 26 | Diseases of the skin and  subcutaneous tissue | L00-L99 |
| 27 | Depression | F32, F33 |
| 28 | Fracture/Dislocation/Sequela | S02, S12, S22, S32, S42, S52, S62, S72, S82, S92, T02, T08, T10, T12, T14.2, T14.3, X59, Y85, Y86, Y87, Y88, Y89 |
| 29 | Obesity | E66 |
| 30 | Purine, pyrimidine metabolism disorders/Gout | E79, M10 |
| 31 | Dementia | F00, F01, F02, F03, F05.1, G30, G31, R54 |
| 32 | Tobacco abuse | F17 |
| 33 | Anxiety | F40, F41 |
| 34 | Somatoform disorders | F45 |
| 35 | Sexual dysfunction | F52, N48.4 |
| 36 | Parkinson’s disease | G20, G21, G22 |
| 37 | Migraine/chronic headache | G43, G44 |
| 38 | Insomnia | G47, F51 |
| 39 | Neuropathies | G50, G51, G52, G53, G54, G55, G56, G57, G58, G59, G60, G61, G62, G63, G64 |
| 40 | Allergies | J30, K52.2, K90.0, T78.1, T78.4, T88.7 |
| 41 | Severe vision reduction | H17, H18, H27, H31, H33, H34.1, H34.2, H34.8, H34.9, H35, H36, H43, H47, H54 |
| 42 | Dizziness | H81, H82, R42 |
| 43 | Severe hearing loss | H90, H91.0, H91.1, H91.3, H91.8, H91.9 |
| 44 | Atherosclerosis/PAOD | I65, I66, I67, I70, I73 |
| 45 | Lower limb varicosis | I83, I87.2 |
| 46 | Hemorrhoids | I84 |
| 47 | Hypotension | I95 |
| 48 | Intestinal diverticulosis | K57 |
| 49 | Chronic cholecystitis/Gallstones | K80, K81.1 |
| 50 | Urinary tract calculi | N20 |
| 51 | Noninflammatory gynecological  problems | N81, N84, N85, N86, N87, N88, N89, N90, N93, N95 |
