# Supplementary material for: Prevalence and Predictors of Polypharmacy among Korean Elderly
Source: PLoS One. 2014 Jun 10;9(6):e98043. doi: 10.1371/journal.pone.0098043 (PMC4051604; doi:10.1371/journal.pone.0098043)
Supplement: Table S2 — The number of drug users in ATC Classification according to polypharmay status in elderly. (DOCX) [file pone.0098043.s002.docx]

**Table S2. The number of drug users in ATC Classification according to polypharmay status in elderly**

| **ATC Classification** | | **Non-Polypharmacy** |  | **Polypharmacy** |  | **Major Polypharmacy** |  | **Excessive Polypharmacy** |
| --- | --- | --- | --- | --- | --- | --- | --- | --- |
|  | | (N = 43,304) |  | (N = 275,881) |  | (N = 143,218) |  | (N = 9,669) |
| **code** | **name** | **Number (%)** |  | **Number (%)** |  | **Number (%)** |  | **Number (%)** |
| A01 | Stomatological preparations | 233 (0.5) |  | 10,203 (3.7) |  | 7,280 (5.1) |  | 936 (9.7) |
| A02 | Drugs for acid related disorders | 18,733 (43.3) |  | 248,632 (90.1) |  | 137,617 (96.1) |  | 9,612 (99.4) |
| A03 | Drugs for functional gastrointestinal disorders | 14,975 (34.6) |  | 227,633 (82.5) |  | 129,805 (90.6) |  | 9,425 (97.5) |
| A04 | Antiemetics and antinauseants | 1,001 (2.3) |  | 35,416 (12.8) |  | 24,775 (17.3) |  | 2,770 (28.6) |
| A05 | Bile and liver therapy | 1,320 (3.0) |  | 33,708 (12.2) |  | 22,450 (15.7) |  | 2,340 (24.2) |
| A06 | Drugs for constipation | 1,039 (2.4) |  | 29,340 (10.6) |  | 20,922 (14.6) |  | 2,830 (29.3) |
| A07 | Antidiarrheals, intestinal antiinflammatory/antiinfective agents | 3,289 (7.6) |  | 89,978 (32.6) |  | 59,269 (41.4) |  | 5,900 (61.0) |
| A09 | Digestives, incl. enzymes | 388 (0.9) |  | 11,933 (4.3) |  | 7,850 (5.5) |  | 793 (8.2) |
| A10 | Drugs used in diabetes | 2,844 (6.6) |  | 59,189 (21.5) |  | 42,428 (29.6) |  | 4,429 (45.8) |
| A11 | Vitamins | 221 (0.5) |  | 7,818 (2.8) |  | 6,034 (4.2) |  | 993 (10.3) |
| A12 | Mineral supplements | 1,053 (2.4) |  | 29,508 (10.7) |  | 20,208 (14.1) |  | 2,046 (21.2) |
| A14 | Anabolic agents for systemic use | 3 (0.0) |  | 13 (0.0) |  | 10 (0.0) |  | 3 (0.0) |
| A15 | Appetite stimulants | 0 (0.0) |  | 76 (0.0) |  | 57 (0.0) |  | 10 (0.1) |
| A16 | Other alimentary tract and metabolism products | 241 (0.6) |  | 12,255 (4.4) |  | 9,602 (6.7) |  | 1,316 (13.6) |
| B01 | Antithrombotic agents | 5,883 (13.6) |  | 116,955 (42.4) |  | 80,197 (56.0) |  | 7,221 (74.7) |
| B02 | Antihemorrhagics | 103 (0.2) |  | 3,217 (1.2) |  | 2,174 (1.5) |  | 275 (2.8) |
| B03 | Antianemic preparations | 439 (1.0) |  | 16,071 (5.8) |  | 12,142 (8.5) |  | 1,820 (18.8) |
| B05 | Blood substitutes and perfusion solutions | 2,124 (4.9) |  | 64,989 (23.6) |  | 46,262 (32.3) |  | 5,529 (57.2) |
| C01 | Cardiac therapy | 1,008 (2.3) |  | 39,130 (14.2) |  | 29,930 (20.9) |  | 3,748 (38.8) |
| C02 | Antihypertensives | 367 (0.8) |  | 8,258 (3.0) |  | 5,798 (4.0) |  | 806 (8.3) |
| C03 | Diuretics | 2,586 (6.0) |  | 60,801 (22.0) |  | 42,753 (29.9) |  | 4,494 (46.5) |
| C04 | Peripheral vasodilators | 2,296 (5.3) |  | 67,646 (24.5) |  | 47,143 (32.9) |  | 4,907 (50.7) |
| C05 | Vasoprotectives | 1,163 (2.7) |  | 34,420 (12.5) |  | 23,163 (16.2) |  | 2,480 (25.6) |
| C07 | Beta blocking agents | 2,886 (6.7) |  | 57,986 (21.0) |  | 40,613 (28.4) |  | 4,130 (42.7) |
| C08 | Calcium channel blockers | 8,021 (18.5) |  | 110,993 (40.2) |  | 69,505 (48.5) |  | 5,649 (58.4) |
| C09 | Agents acting on the renin-angiotensin system | 6,744 (15.6) |  | 106,457 (38.6) |  | 70,317 (49.1) |  | 6,183 (63.9) |
| C10 | Lipid modifying agents | 3,169 (7.3) |  | 76,751 (27.8) |  | 54,209 (37.9) |  | 5,297 (54.8) |
| D01 | Antifungals for dermatological use | 2,138 (4.9) |  | 40,927 (14.8) |  | 26,263 (18.3) |  | 2,652 (27.4) |
| D02 | Emollients and protectives | 163 (0.4) |  | 3,125 (1.1) |  | 2,104 (1.5) |  | 241 (2.5) |
| D03 | Preparations for treatment of wounds and ulcers | 74 (0.2) |  | 1,146 (0.4) |  | 755 (0.5) |  | 87 (0.9) |
| D04 | Antipruritics, incl. antihistamines, anesthetics, etc. | 0 (0.0) |  | 41 (0.0) |  | 24 (0.0) |  | 4 (0.0) |
| D05 | Antipsoriatics | 62 (0.1) |  | 1,180 (0.4) |  | 812 (0.6) |  | 99 (1.0) |
| D06 | Antibiotics and chemotherapeutics for dermatological use | 919 (2.1) |  | 20,425 (7.4) |  | 13,444 (9.4) |  | 1,466 (15.2) |
| D07 | Corticosteroids, dermatological preparations | 3,706 (8.6) |  | 69,596 (25.2) |  | 42,644 (29.8) |  | 3,839 (39.7) |
| D08 | Antiseptics and disinfectants | 13 (0.0) |  | 510 (0.2) |  | 371 (0.3) |  | 53 (0.5) |
| D10 | Anti-acne preparations | 0 (0.0) |  | 27 (0.0) |  | 19 (0.0) |  | 4 (0.0) |
| D11 | Other dermatological preparations | 43 (0.1) |  | 1,850 (0.7) |  | 1,367 (1.0) |  | 208 (2.2) |
| G01 | Gynecological antiinfectives and antiseptics | 210 (0.5) |  | 5,696 (2.1) |  | 3,797 (2.7) |  | 386 (4.0) |
| G02 | Other gynecologicals | 5 (0.0) |  | 188 (0.1) |  | 135 (0.1) |  | 16 (0.2) |
| G03 | Sex hormones and modulators of the genital system | 423 (1.0) |  | 9,364 (3.4) |  | 6,234 (4.4) |  | 635 (6.6) |
| G04 | Urologicals | 2,221 (5.1) |  | 45,207 (16.4) |  | 30,803 (21.5) |  | 3,607 (37.3) |
| H01 | Pituitary and hypothalamic hormones and analogues | 67 (0.2) |  | 2,061 (0.7) |  | 1,581 (1.1) |  | 263 (2.7) |
| H02 | Corticosteroids for systemic use | 6,173 (14.3) |  | 145,967 (52.9) |  | 89,455 (62.5) |  | 7,565 (78.2) |
| H03 | Thyroid therapy | 323 (0.7) |  | 6,517 (2.4) |  | 4,537 (3.2) |  | 505 (5.2) |
| H04 | Pancreatic hormones | 0 (0.0) |  | 9 (0.0) |  | 8 (0.0) |  | 1 (0.0) |
| H05 | Calcium homeostasis | 104 (0.2) |  | 3,155 (1.1) |  | 2,152 (1.5) |  | 235 (2.4) |
| J01 | Antibacterials for systemic use | 9,304 (21.5) |  | 190,964 (69.2) |  | 112,591 (78.6) |  | 8,746 (90.5) |
| J02 | Antimycotics for systemic use | 1,319 (3.0) |  | 23,723 (8.6) |  | 15,180 (10.6) |  | 1,559 (16.1) |
| J04 | Antimycobacterials | 23 (0.1) |  | 931 (0.3) |  | 699 (0.5) |  | 86 (0.9) |
| J05 | Antivirals for systemic use | 500 (1.2) |  | 12,224 (4.4) |  | 7,820 (5.5) |  | 762 (7.9) |
| J06 | Immune sera and immunoglobulins | 97 (0.2) |  | 3,395 (1.2) |  | 1,969 (1.4) |  | 192 (2.0) |
| J07 | Vaccines | 0 (0.0) |  | 3 (0.0) |  | 2 (0.0) |  | 0 (0.0) |
| L01 | Antineoplastic agents | 24 (0.1) |  | 1,406 (0.5) |  | 1,138 (0.8) |  | 168 (1.7) |
| L02 | Endocrine therapy | 20 (0.0) |  | 665 (0.2) |  | 525 (0.4) |  | 103 (1.1) |
| L03 | Immunostimulants | 31 (0.1) |  | 1,107 (0.4) |  | 818 (0.6) |  | 128 (1.3) |
| L04 | Immunosuppressants | 10 (0.0) |  | 873 (0.3) |  | 721 (0.5) |  | 131 (1.4) |
| M01 | Antiinflammatory and antirheumatic products | 17,388 (40.2) |  | 232,973 (84.4) |  | 129,987 (90.8) |  | 9,289 (96.1) |
| M02 | Topical products for joint and muscular pain | 382 (0.9) |  | 10,378 (3.8) |  | 6,880 (4.8) |  | 739 (7.6) |
| M03 | Muscle relaxants | 6,412 (14.8) |  | 127,189 (46.1) |  | 78,274 (54.7) |  | 6,557 (67.8) |
| M04 | Antigout preparations | 181 (0.4) |  | 5,123 (1.9) |  | 3,596 (2.5) |  | 491 (5.1) |
| M05 | Drugs for treatment of bone diseases | 1,722 (4.0) |  | 38,675 (14.0) |  | 25,818 (18.0) |  | 2,589 (26.8) |
| M09 | Other drugs for disorders of the musculo-skeletal system | 9,823 (22.7) |  | 174,640 (63.3) |  | 102,245 (71.4) |  | 7,882 (81.5) |
| N01 | Anesthetics | 1,940 (4.5) |  | 62,150 (22.5) |  | 42,216 (29.5) |  | 4,437 (45.9) |
| N02 | Analgesics | 13,156 (30.4) |  | 218,292 (79.1) |  | 124,949 (87.2) |  | 9,226 (95.4) |
| N03 | Antiepileptics | 860 (2.0) |  | 29,194 (10.6) |  | 22,195 (15.5) |  | 3,198 (33.1) |
| N04 | Anti-parkinson drugs | 303 (0.7) |  | 7,525 (2.7) |  | 5,806 (4.1) |  | 973 (10.1) |
| N05 | Psycholeptics | 5,841 (13.5) |  | 141,187 (51.2) |  | 91,765 (64.1) |  | 8,177 (84.6) |
| N06 | Psychoanaleptics | 3,173 (7.3) |  | 72,468 (26.3) |  | 51,881 (36.2) |  | 6,031 (62.4) |
| N07 | Other nervous system drugs | 819 (1.9) |  | 22,918 (8.3) |  | 16,732 (11.7) |  | 2,194 (22.7) |
| P01 | Antiprotozoals | 36 (0.1) |  | 2,126 (0.8) |  | 1,679 (1.2) |  | 239 (2.5) |
| P02 | Anthelmintics | 9 (0.0) |  | 164 (0.1) |  | 102 (0.1) |  | 6 (0.1) |
| P03 | Ectoparasiticides, incl. scabicides, insecticides and repellents | 103 (0.2) |  | 1,335 (0.5) |  | 852 (0.6) |  | 83 (0.9) |
| R01 | Nasal preparations | 2,274 (5.3) |  | 77,522 (28.1) |  | 48,821 (34.1) |  | 4,362 (45.1) |
| R02 | Throat preparations | 336 (0.8) |  | 19,538 (7.1) |  | 14,308 (10.0) |  | 1,762 (18.2) |
| R03 | Drugs for obstructive airway diseases | 2,072 (4.8) |  | 84,230 (30.5) |  | 56,085 (39.2) |  | 5,680 (58.7) |
| R05 | Cough and cold preparations | 9,089 (21.0) |  | 196,988 (71.4) |  | 114,338 (79.8) |  | 8,723 (90.2) |
| R06 | Antihistamines for systemic use | 9,581 (22.1) |  | 190,269 (69.0) |  | 110,345 (77.0) |  | 8,553 (88.5) |
| R07 | Other respiratory system products | 132 (0.3) |  | 5,681 (2.1) |  | 3,970 (2.8) |  | 500 (5.2) |
| S01 | Ophthalmologicals | 10,030 (23.2) |  | 133,030 (48.2) |  | 80,233 (56.0) |  | 6,760 (69.9) |
| S02 | Otologicals | 347 (0.8) |  | 4,864 (1.8) |  | 3,055 (2.1) |  | 296 (3.1) |
| S03 | Ophthalmological and otological preparations | 19 (0.0) |  | 433 (0.2) |  | 285 (0.2) |  | 31 (0.3) |
| V03 | All other therapeutic products | 240 (0.6) |  | 5,402 (2.0) |  | 4,297 (3.0) |  | 924 (9.6) |
| V04 | Diagnostic agents | 21 (0.0) |  | 406 (0.1) |  | 286 (0.2) |  | 31 (0.3) |
| V06 | General nutrients | 8 (0.0) |  | 175 (0.1) |  | 140 (0.1) |  | 26 (0.3) |
| V07 | All other non-therapeutic products | 110 (0.3) |  | 7,298 (2.6) |  | 5,314 (3.7) |  | 721 (7.5) |
| V08 | Contrast media | 913 (2.1) |  | 20,948 (7.6) |  | 14,745 (10.3) |  | 1,862 (19.3) |
| V09 | Diagnostic radiopharmaceuticals | 75 (0.2) |  | 1,866 (0.7) |  | 1,311 (0.9) |  | 149 (1.5) |
| V10 | Therapeutic radiopharmaceuticals | 0 (0.0) |  | 1 (0.0) |  | 0 (0.0) |  | 0 (0.0) |
